# Triboelectric Nanogenerators Based on Transition Metal Carbo‐Chalcogenide (Nb_2_S_2_C and Ta_2_S_2_C) for Energy Harvesting and Self‐Powered Sensing

**DOI:** 10.1002/advs.202409619

**Published:** 2024-09-25

**Authors:** Yana Xiao, Zihua Li, Di Tan, Gachot Carsten, Bingang Xu

**Affiliations:** ^1^ Nanotechnology Center School of Fashion and Textiles The Hong Kong Polytechnic University Hung Hom Kowloon Hong Kong 999077 P. R. China; ^2^ Institute for Engineering Design and Product Development Tribology Research Division TU Wien Leh´argasse 6 Objekt 7 Vienna 1060 Austria

**Keywords:** coefficient of friction, energy harvesting, transition metal carbo‐chalcogenides, triboelectric nanogenerator, tribological properties

## Abstract

With burgeoning considerations over energy issues and carbon emissions, energy harvesting devices such as triboelectric nanogenerators (TENGs) are developed to provide renewable and sustainable power. Enhancing electric output and other properties of TENGs during operation is the focus of research. Herein, two species (Nb_2_S_2_C and Ta_2_S_2_C) of a new family of 2D materials, Transition Metal Carbo‐Chalcogenides (TMCCs), are first employed to develop TENGs with doping into Polydimethylsiloxane (PDMS). Compared with control samples, these two TMCC‐based TENGs exhibit higher electric properties owing to the enhanced permittivity of PDMS composite, and the best performance is achieved at a concentration of 3 wt. ‰ with open circuit voltage (Voc) of 112 V, short circuit current (Isc) of 8.6 µA and charge transfer (Qsc) of 175 nC for Nb_2_S_2_C based TENG, and Voc of 127 V, Isc of 9.6 µA, and Qsc of 230 nC for Ta_2_S_2_C based TENGs. These two TENGs show a maximum power density of 1360 and 911 mW m^−2^ respectively. Moreover, the tribology performance is also evaluated with the same materials, revealing that the Ta_2_S_2_C/PDMS composite as the electronegative material presented a lower coefficient of friction (COF) than the Nb_2_S_2_C/PDMS composite. Their applications for energy harvesting and self‐powered sensing are also demonstrated.

## Graphical abstract

Transition metal carbo‐chalcogenide (TMCC), Nb_2_S_2_C and Ta_2_S_2_C, are first employed to fabricate triboelectric nanogenerator (TENG) with polydimethylsiloxane (PDMS).TMCC‐TENGs exhibit enhanced electrical properties with optimum value both at 3 wt.‰. Maximum power density reaches 1360 mW m^−2^ for Nb_2_S_2_C‐TENG and 911 mW m^−2^ for Ta_2_S_2_C‐TENG. Ta_2_S_2_C/PDMS composite exhibits reduced COF compared to Nb_2_S_2_C/PDMS.

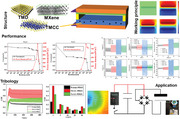

## 1 Introduction

Considering the massive consumption of fossil fuels and environmental issues due to traditional batteries, along with the rapid growth of various wearable devices and Internet‐of‐Things (IoT) enabled devices, harvesting available renewable energy sources is essential to healing current energy dependence.^[^ ^1^ ^]^ Sustainable and net‐zero carbon emission power sources are acting as a thriving factor due to booming energy consumption. Energy harvesting based on nanogenerators has played a significant role in advancing clean energy.^[^ ^2^ ^]^ The energy harvesting nanogenerators such as piezoelectric nanogenerators (PENG),^[^ ^3^ ^]^ pyroelectric nanogenerators (PyNGs), moisture electric generators (MEGs),^[^ ^4^ ^]^ triboelectric nanogenerators (TENGs),^[^ ^5^ ^]^ tribovotaic nanogenerators (TVNGs),^[^ ^6^ ^]^ or electromagnetic generators (EMGs) have come into play to harness and convert environmental energies like mechanical, thermal and solar energy sustainably and efficiently into electrical energy.^[^ ^7^, ^8^ ^]^ Particularly, TENGs are attractive owing to their low costs, mechanical flexibility,^[^ ^9^ ^]^ easy assembly, and their low‐frequency working range.^[^ ^10^ ^]^

For TENGs,^[^ ^11^ ^]^ two kinds of material with different electronegativities and electrodes are used, and the first TENGs were made with polyethylene terephthalate (PET) and polyimide (PI).^[^ ^12^, ^13^ ^]^ Nowadays, Polydimethylsiloxane (PDMS) is often employed, which is a bio‐friendly, non‐toxic, flexible, highly durable, translucent, low‐cost, and highly electronegative material. However, polymers usually obtain low dielectric constant as well as low capacitance and low charge abstaining capacity^[^ ^14^ ^]^ accordingly, especially on TENGs during application with other electronic devices^[^ ^15^ ^]^ as both energy harvesters and self‐powered sensors.^[^ ^16^ ^]^ So far, major strategies implemented for improving TENG's electrical output have aimed at enhancing the triboelectric material dielectric performance^[^ ^17^, ^18^ ^]^ as well as the accumulated charge transfer density.^[^ ^19^, ^20^ ^]^ The introduction of high permittivity particles^[^ ^21^ ^]^ into the substrate^[^ ^22^, ^23^ ^]^ at the interface, between the triboelectric materials^[^ ^24^ ^]^ or with the electrode^[^ ^25^, ^26^, ^27^ ^]^ has been proven effective in increasing the performance of TENG, from a wide variety of carbon nanotubes,^[^ ^28^ ^]^ ferromagnetic barium titanate,^[^ ^29^ ^]^ various 2D materials,^[^ ^30^, ^31^ ^]^ to liquid metals,^[^ ^32^ ^]^ etc.

In addition to the need to improve the electric properties of TENGs, research has shown that their tribological properties are of paramount importance as well.^[^ ^33^ ^]^ Specifically, their wear resistance during sliding is a crucial factor in limiting their applicability.^[^ ^34^ ^]^ For example, by adding PAO_4_ as a lubricant, the coefficient of friction (COF) of the TCDC‐TENG^[^ ^35^ ^]^ was lowered. Moreover, water‐based graphene oxide could be utilized as a lubricant to enhance current density and reduce wear.^[^ ^36^ ^]^ In this context, the addition of specifically 2D materials to the substrate matrix can have positive effects on the wear behavior^[^ ^37^ ^]^ apart from improving the electric performance of TENGs.^[^ ^38^ ^]^

2D materials have a commonality in their electrical production properties owing to their specificity in electron transport, which is another breakthrough for our research on performance enhancement and the commonality of 2D materials. 2D materials with special structures and properties at the level of a few atoms or molecules in thickness have been widely studied in recent years including graphene/graphene oxide,^[^ ^38^ ^]^ transition metal dichalcogenides (TMDs),^[^ ^39^ ^]^ MXene,^[^ ^40^ ^]^ carbon nitride,^[^ ^30^ ^]^ transition metal carbo‐chalcogenide (TMCC),^[^ ^41^ ^]^ etc. These materials have attracted much attention in the fields of electronics, optics, and energy owing to their unique characteristic properties.^[^ ^42^ ^]^ MXenes belonging to the family of transition metal carbides and nitrides^[^ ^43^, ^44^ ^]^ are well‐known for their good chemical stability, high mechanical strength as well as excellent electrical conductivity. The two most used TMDs are molybdenum disulfide (MoS_2_) and tungsten disulfide (WS_2_) with different electronic band structures^[^ ^45^ ^]^ and polymorphic structures.^[^ ^46^ ^]^ The single‐layered TMCC of these two species (Nb_2_S_2_C and Ta_2_S_2_C) was first successfully realized from multilayered Nb_2_S_2_C^[^ ^47^ ^]^ and Ta_2_S_2_C through electrochemical lithiation and sonication in 2022 and it was experimentally verified that the delaminated Nb_2_S_2_C outperformed its multilayered precursor material as an electrode material in electrochemistry.^[^ ^41^ ^]^

TMCC can be considered an atomic combination of transition metal carbonitrides (MXene)^[^ ^48^ ^]^ and transition metal dichalcogenides (TMDs),^[^ ^10^ ^]^ which could be obtained by combining one layer of MXene with two half‐layers of TMD.^[^ ^49^ ^]^ The nature of the interaction between those 2D materials could contribute greatly to applications of energy storage,^[^ ^49^ ^]^ especially to the enhancement of TENG's performance demonstrated by plenty of previous research with TMD^[^ ^10^ ^]^ and MXene.^[^ ^50^, ^51^, ^52^ ^]^ It is expected that TMCC as their combination at the atomic level, would have a positive role in improving the electric performance of TENGs. However, to our best knowledge, there is no such research so far, not to mention the combination of tribology and triboelectric performance.

Therefore, in this research, two new species (Nb_2_S_2_C and Ta_2_S_2_C) of Transition Metal Carbo‐Chalcogenides (TMCCs) were originally employed to develop TENGs doping with PDMS. In terms of the enhancement of electrical performance, both Nb_2_S_2_C‐based TENG and Ta_2_S_2_C‐based TENG were comparable, achieving the best electrical performance at the concentration of 3 wt.‰. In addition, the tribological properties were investigated on a ball‐on‐disk setup against a steel ball counter body. Ta_2_S_2_C/PDMS composite as the electronegative material was softer and presented a lower COF than pristine PDMS and Nb_2_S_2_C/PDMS composite. Their applications for energy harvesting and self‐powered sensing were also demonstrated.

## 2 Results and Discussion

### 2.1 Fabrication, Mechanism and Characterization

**Figure 1:**
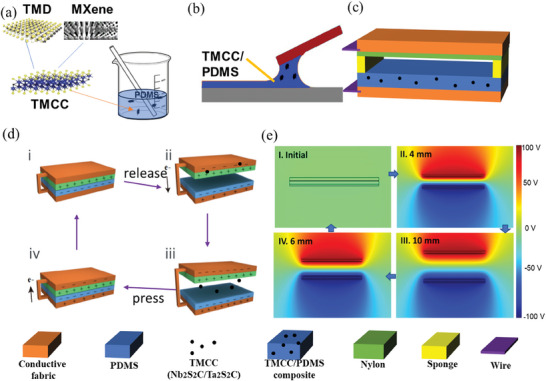
Fabrication process of a) Doping b) Blade coating for TMCC/PDMS composites. c) The architecture of the vertical contact‐separation mode of TENG d) Mechanism illustration and e) COMSOL simulation for a cycle of contact‐separation.

Monolayer Nb_2_S_2_C has a molecular structure where one layer of carbon atoms is sandwiched by two layers of Nb/Ta atoms, with a layer of S atoms above and below the outermost layer, while NbS_2_ has a molecular structure where two hexagonal S atomic layers sandwich a layer of transition metal atoms, as shown in **Figure** **1**a. Therefore, 2D layers of Nb_2_S_2_C can combine the chemical reactivity of NbS_2_ while maintaining a mechanically robust and metallically conductor carbide core which is not achievable by transition metal carbide or nitride alone. The other TMCC adopted in this study Ta_2_S_2_C has similar structure and properties.

The creation of TMCC/PDMS composites involved the incorporation of TMCC particles into a PDMS precursor as depicted in Figure 1a, followed by a blading process in Figure 1b. After curing, the TMCC/PDMS composite was then constructed into a contact‐separation mode TENG, as demonstrated in Figure 1c. Here, nylon served as the positive dielectric, while the TMCC/PDMS composite functions as the negative dielectric. Both dissimilar dielectric films were attached to Cu/Ni conductive fabric (CNF) as electrodes on each side with the other side opposite to each other. We chose Cu/Ni conductive fabric as the electrode because the Cu/Ni conductive fabric has good flexibility and air permeability as well as strong conductivity, which is very suitable for wearable electronics. The operational mechanism of the vertical contact‐separation mode under an external force is schematically represented, demonstrating the electron transfer process in Figure 1d. Due to their varying electron affinities, an equal number of positive and negative charges were generated on the surface of both dielectric layers in the initial state (Figure 1d‐i). In the second state (Figure 1d‐ii), the external force separated the two membranes, creating a gap and an electric potential difference, thus generating an electrical current with electron transfer from one electrode to the other via an external load (or electrometer). When the two layers were fully separated (Figure 1d‐iii), electrostatic equilibrium was achieved, and no electron movement occurred. When the external force disrupted this equilibrium, a new opposite potential difference was created, causing the electron current to flow in the opposite direction (Figure 1d‐iv). Besides electron transfer where orbital overlap leads to charge tunneling, there is also mass (material) transfer where polymer chain entanglement and intermolecular bonding lead to heterolytic bond scission,^[^ ^53^ ^]^ as well as ion transfer.^[^ ^54^, ^55^ ^]^ Throughout the contact and separation process, electrons continuously flowed between the bottom and top electrodes, generating alternating current (AC).^[^ ^56^ ^]^ For an understanding, the associated potential distribution of the positive and negative dielectric materials was simulated and displayed using COMSOL Multiphysics software, as shown in Figure 1e.

**Figure 2:**
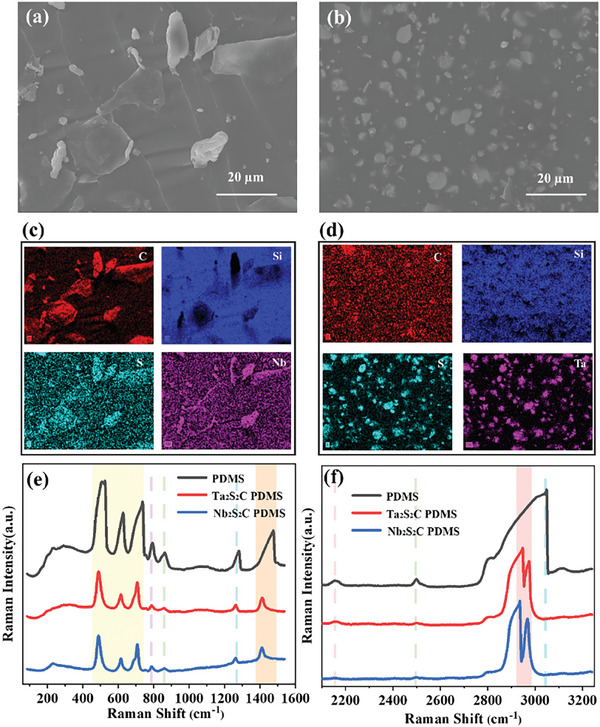
Morphology analysis and structural characteristics of tribo‐layer materials. a) SEM images of Nb_2_S_2_C/PDMS composite samples. b) SEM images of Ta_2_S_2_C/PDMS composite samples. c) EDS‐elemental mapping images of the Nb_2_S_2_C/PDMS composite sample. d) EDS‐elemental mapping images of the Ta_2_S_2_C/PDMS composite sample. e,f) Raman pattern of pure PDMS, Nb_2_S_2_C/PDMS composite, and Ta_2_S_2_C/PDMS composite.

From the scanning electron microscopy (SEM) images of Nb_2_S_2_C/PDMS composite film in **Figure** **2**a and Ta_2_S_2_C/PDMS composite in Figure 2b, it could be seen that Nb_2_S_2_C and Ta_2_S_2_C particles were dispersed into the PDMS matrix. The corresponding EDS‐elemental mapping images authenticated the obvious presence of Niobium (Figure 2c) or Tantalum (Figure 2d) elements. As silicon was only present in PDMS but not in the dopants, the silicon element was shaded in the corresponding EDS image where the transition metal, carbon, and sulfur elements were present, indicating that TMCC/PDMS composites were successfully prepared.

Raman spectroscopy in Figure 2e–f can detect the fingerprint pattern and quantitatively be employed to identify phases. The Raman spectroscopy showed peaks at wavelengths of ≈2,960 and 2 970 cm^−1^ in Nb2S2C/PDMS composites and Ta2S2C/PDMS composites respectively, which were absent as compared to pure PDMS. Moreover, other Raman characteristic peaks of PDMS at ≈490, ≈600, ≈700, ≈1 280, ≈1 420, ≈2 500 cm^−1^ became less significant after doping of TMCC particles.

X‐ray photoelectron spectroscopy (XPS) was utilized to study the surface chemistry of the Nb_2_S_2_C/PDMS composite, as shown in Figure S2a–d (Supporting Information), as well as the Ta_2_S_2_C/PDMS composite shown in Figure S2e–h (Supporting Information). The Ta 4f spectrum at ≈24 eV was assigned to Ta_2_S_2_C. Comparatively, the Nb 3d spectrum at ≈203 eV assigned to Nb_2_S_2_C was not that obvious. C elements had significant existence in both TMCC and PDMS substrates, and S element also showed for C‐Nb‐S or C‐Ta‐S.

More EDS mapping was shown in Figure S1a,b (Supporting Information), and X‐ray diffraction patterns were listed in Figure S1c,d (Supporting Information). The diffraction peaks at 2θ ≈12° and ≈13° were attributed to doping of Nb_2_S_2_C and Ta_2_S_2_C, respectively. The dielectric constant plays a crucial role in determining the electric performance of TENGs, and the influence of dopants on the dielectric properties of PDMS was examined. PDMS films of 4 cm^2^ were encapsulated on both sides of conductive plates to create a parallel plate capacitance model. The capacitance values of all samples were measured across a frequency range of 10^2^ to 2MHz. The method for measuring the dielectric constant is based on the parallel plate capacitor conditions, and the dielectric constant can be calculated using the following formula:

(1) C=εS/4πkd

In the formula above, *C* stands for capacitance, *ε* symbolizes the dielectric constant, *k* is the electrostatic force constant (*k* = 8.9880 × 10^9^ Nm C^−1^), *d* represents the thickness of the film (or the distance between two electrode plates), and *S* refers to the effective area (or overlapping surface area) of two electrode plates.^[^ ^23^ ^]^ The dielectric constant (or permittivity) was related to the contents of the dopant in the PDMS substrate. The dielectric constant slightly increased by about 2% when the ratio of TMCC doped into pure PDMS from 1 to 3 wt.‰, while excessive amount of TMCC dopants in PDMS up to 10 wt.‰ could decrease the dielectric constant by of TMCC composite to around 3.12 which was about the same at 1 wt.‰, for both Nb_2_S_2_C (Figure S1e, Supporting Information) and Ta_2_S_2_C (Figure S1f, Supporting Information). This is consistent with the electrical output trend at different concentrations, confirming that a higher dielectric constant results in more electrostatic energy storage capacity in an electric field and thus better performance of TENG.^[^ ^22^ ^]^

Surface charge density is one of the key performance indicators of triboelectric nanogenerators (TENGs) and is a critical parameter for measuring their power generation efficiency. Upon comparison, it was found that the 2D material/PDMS composite materials exhibit a significantly higher surface charge density, which will be discussed in the following section.

### 2.2 Electrical Output

**Figure 3:**
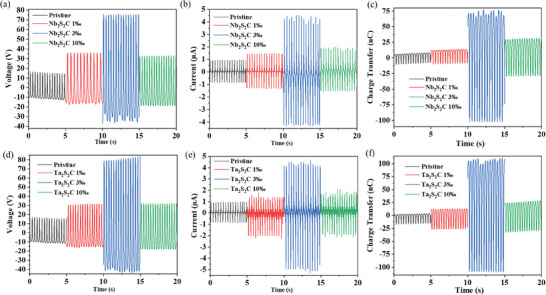
Electric performance at 40 N, 3 Hz, 16 cm^2^ a) Open circuit voltage, b) Short circuit current, c) Charge transfer of Nb_2_S_2_C based TENG at different weight ratios, and d) Open circuit voltage, e) Short circuit current, f) Charge transfer of Ta_2_S_2_C based TENG at different weight ratios.

Both TENGs using tribonegative material of TMCC/PDMS composites (TMCC‐TENGs) were systematically fabricated and evaluated with different weight ratio concentrations of 1‰, 3‰, and 10‰, respectively. To test the electric performance in dependence of the TMCC concentrations, the voltage, current, and charge transfer were measured (**Figure** **3**) in contact separation mode at 20 N and 3 Hz impact, utilizing TENG of 16 cm^2^ size (4 cm × 4 cm). For the pristine PDMS, it is observed an open circuit voltage (Voc) of 25 V, short circuit current (Isc) of 1.8 µA, and charge transfer (Qsc) of 21 nC. Compared with pristine PDMS, TMCC‐TENGs substantially improved the electrical performance regardless of the concentration. The optimum weight ratio to achieve the best electric performance for Nb_2_S_2_C‐based TENG appeared at 3 wt.‰, with open circuit voltage (Voc) of 112 V, short circuit current (Isc) of 8.6 µA, and charge transfer (Qsc) of 175 nC. Similarly, the optimum weight ratio for Ta_2_S_2_C‐based TENG was also 3 wt.‰, reaching a Voc of 127 V, Isc of 9.6 µA, and Qsc of ≈230 nC. This trend indicates that the incorporation of Nb₂S₂C and Ta₂S₂C into PDMS significantly enhances its ability to store charge. The enhancement in TENG performance might be mainly attributed to the polarization of particles with an increase in dielectric constant and charge trapping. The introduced TMCC nanoparticles increased the permittivity of composite and the capacitance of friction materials, and then comprehensively enhanced the output performance,^[^ ^17^, ^21^ ^]^ while over a certain concentration, the permittivity and electrical performance will decrease. Niobium and tantalum are transition metals as indispensable twin elements with shared physical and chemical properties, which is why Nb_2_S_2_C/PDMS and Ta_2_S_2_C/PDMS TENG had similar performances. We further explored the surface charge density through Kelvin Probe Force Microscopy (KPFM), as shown in Figure S4 (Supporting Information). The measured voltage of pristine PDMS is around 40 mV on average, which was higher than either Ta_2_S_2_C/PDMS composite (−20 mV) or Nb_2_S_2_C/PDMS composite (−160 mV), demonstrating that TMCC/PDMS composite is more negative and possesses stronger electron absorption ability. Furthermore, TMCC/PDMS composite materials exhibit a significantly higher surface charge density as calculated by charge transfer, being 1.09375 × 10^−4^ C m^−2^ for Nb_2_S_2_C/PDMS composite and 1.4375 × 10^−4 ^C m^−2^ for Ta_2_S_2_C/PDMS composite, which is 8.33 times and 10.95 times higher than pristine PDMS, respectively. Thereby higher surface charge density resulted in better electrical performance as well as friction and energy efficiency for TMCC/PDMS composite with identical tribopositive materials.

### 2.3 Impact of External Factors

**Figure 4:**
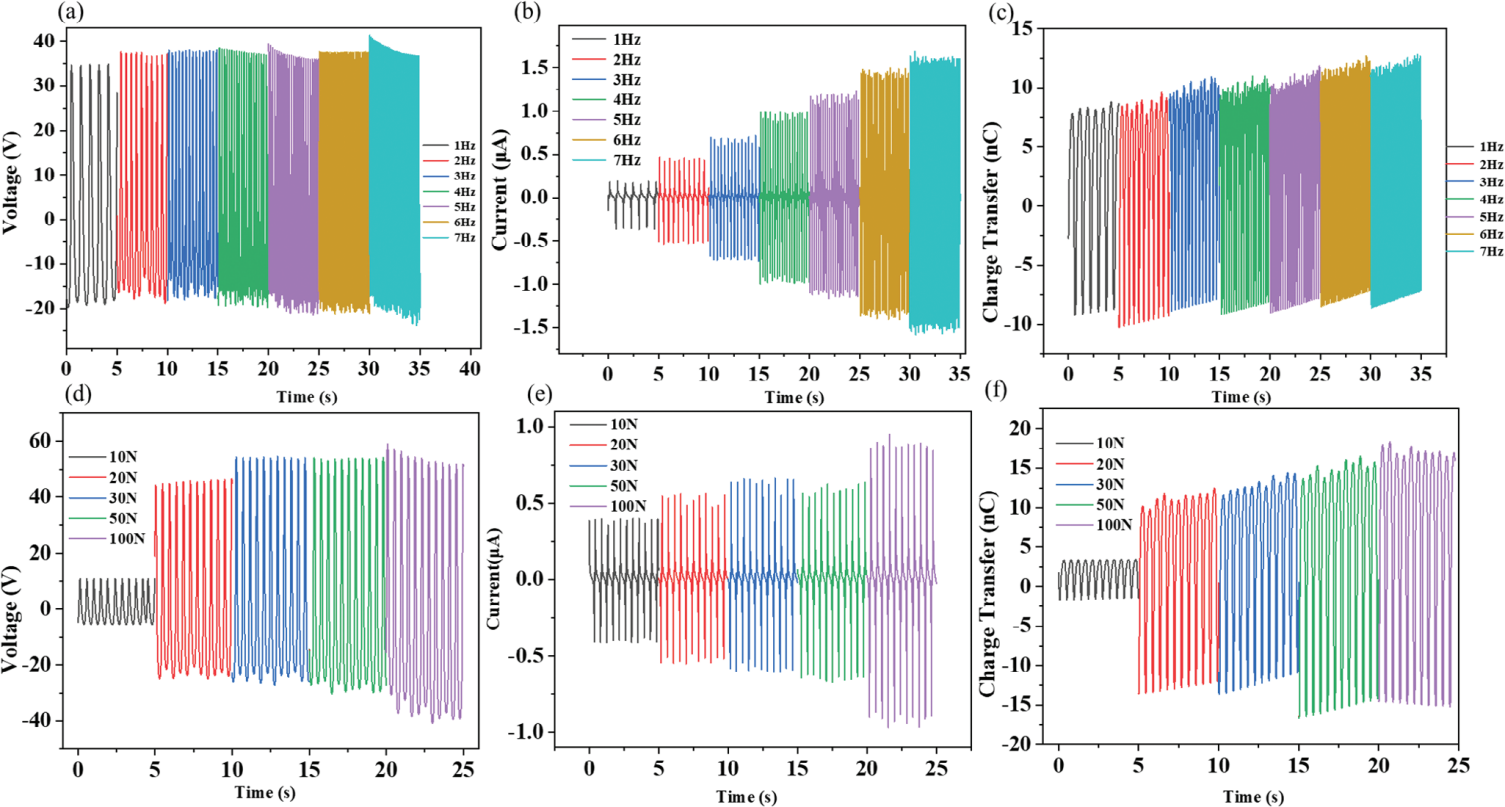
Different frequencies or forces on 3 wt.‰ Nb_2_S_2_C based TENG of 4 cm^2^ size for electrical performance a) Open circuit voltage b) Short circuit current c) Charge transfer at different frequencies from 1 to 7 Hz at a force of 20 N and d) Open circuit voltage e) Short circuit current f) Charge transfer at different impact forces of 10, 20, 30, 50, and 100 N at a frequency of 2 Hz.

**Figure 5:**
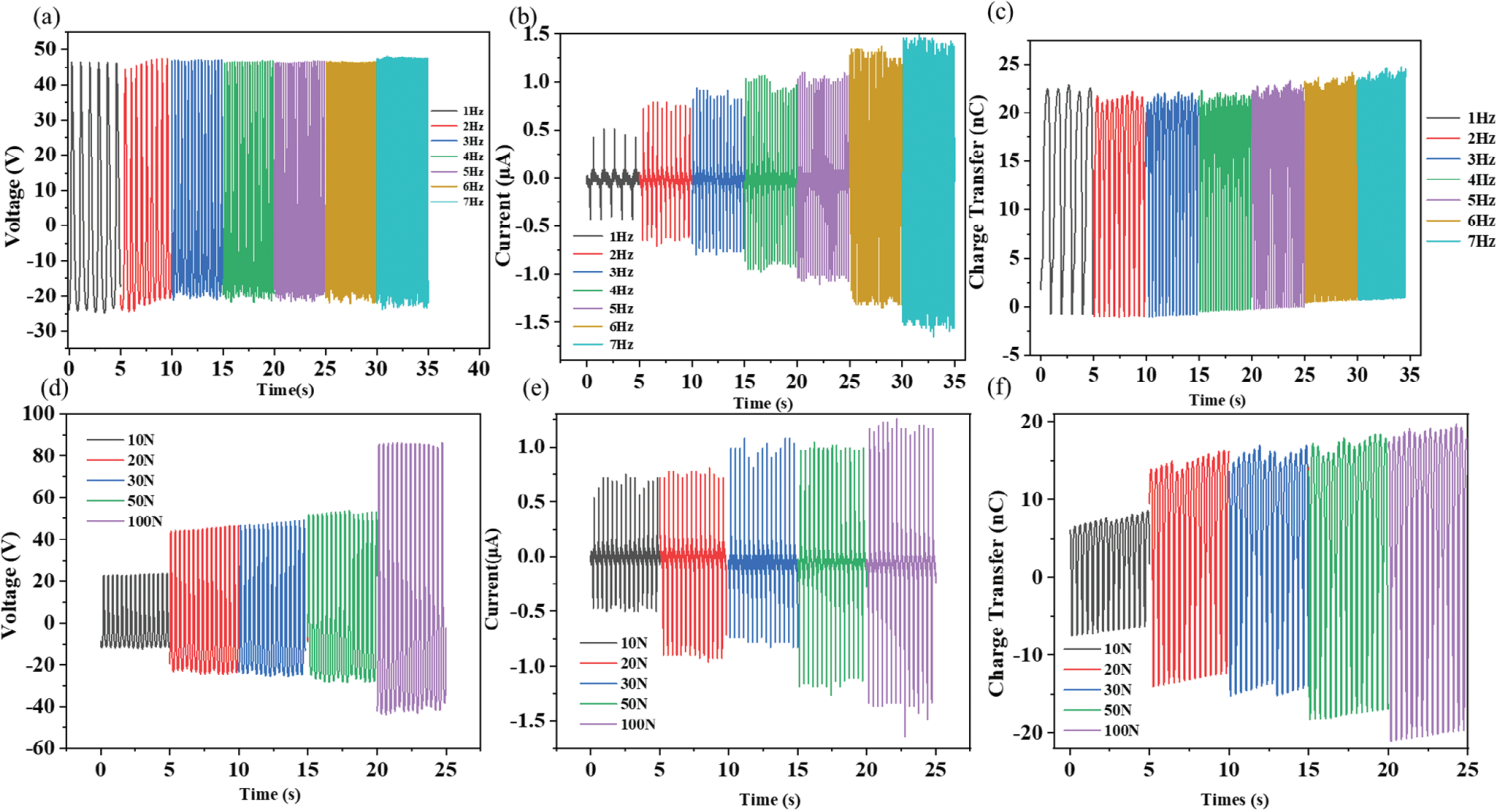
Different frequencies or forces on 3 wt.‰ Ta_2_S_2_C‐based TENG of 4 cm^2^ size for electrical performance a) Open circuit voltage b) Short circuit current c) Charge transfer at different frequencies from 1 to 7 Hz at a force of 20 N and d) Open circuit voltage e) Short circuit current f) Charge transfer at different impact forces of 10, 20, 30, 50, and 100 N.

We have also evaluated the influence of two main external impact factors (force and frequency) on the electrical output performance of both Nb_2_S_2_C‐based TENG (**Figure** **4**) and Ta_2_S_2_C‐based TENG (**Figure** **5**). We adopted Cu/Ni conductive fabric as both positive dielectric and electrode and PDMS composite as negative dielectric with a smaller effective size of 4 cm^2^ (2 cm × 2 cm). Specifically, at a fixed impact force of 20 N, with the frequency increased gradually from 1 to 7 Hz, the Voc increased slightly from 50 to 59 V (Figure 4a), with Isc raised from 0.6 to 3.0 µA (Figure 4b) and Qsc increased from 17 to 20 nC (Figure 4c). While at a set frequency of 2 Hz, when the impact force increased from 10 to 20 N, the Voc and Qsc increased significantly from 15 V/6 nC to 65 V/24 nC, but only increased slightly when further increased the impact force up to 100 N with the maximum value of 90 V/32 nC, as shown in Figure 4d,f, while the Isc increased sequentially from 0.8 to 1.8 µA (Figure 4e), correspondingly to the impact forces from 10 to 100 N.

For the Ta_2_S_2_C‐based TENG, at a fixed impact force of 20 N with a contact‐separation distance of ≈1.7 cm, as the frequency increased gradually from 1 to 7 Hz, the Voc and Qsc remained similarly at around 70 V (Figure 5a)/42 nC (Figure 5c), while the Isc increased constantly from 1.0 to 3.05 µA (Figure 5b). The influence of different impact forces of 10, 20, 30, 50, and 100 N at a fixed frequency of 2 Hz showed a general increase in Voc, Isc, and Qsc, with an approximate increase from 37 to 125 V (Figure 5d), 1.08 to 2.6 µA (Figure 5e), 14 to 39 nC (Figure 5f), respectively.

A greater force leads to a more intimate contact between the two triboelectric surfaces with a larger contact area and more charge transfer. The output voltage and charge transfer of a TENG are directly related to the amount of charge transferred, while the current is approximately proportional to the rate at which this transfer occurs. A greater force represents a higher mechanical energy input into the system, which can lead to a higher output because it enhances the charge transfer process and the rate of change in the electric field, both are crucial for generating electricity in a TENG. At certain impact forces, a higher frequency can stimulate electrons faster and the surface charge of the contact layer cannot be quickly neutralized at an elevated frequency, which generates an enlarged current. Attributed to more intimate contact between positive and negative dielectric, the output of the TENG increases at a larger magnitude of applied impact force.^[^ ^57^ ^]^ The inconsistent variation of electrical outputs Voc/Qsc and Isc with frequency change might be explained according to the Gauss theorem, where the Voc and Qsc are independent of speed, which means that the variation of contact frequency will cause little change of the Voc and Qsc,^[^ ^58^ ^]^ while Isc strictly in positive correlation with the relative movement speed/frequency.^[^ ^59^ ^]^

### 2.4 Applications

Because the contact‐separation mode TENG can only generate an alternating current, a rectifier needs to be used for converting an AC into a direct current (DC). The full‐wave rectifier uses two diodes to pass both the positive and negative halves of the incoming AC wave to the output, resulting in a DC signal, and then capacitors could be charged for powering devices, as shown in Video S1 (Supporting Information).

**Figure 6:**
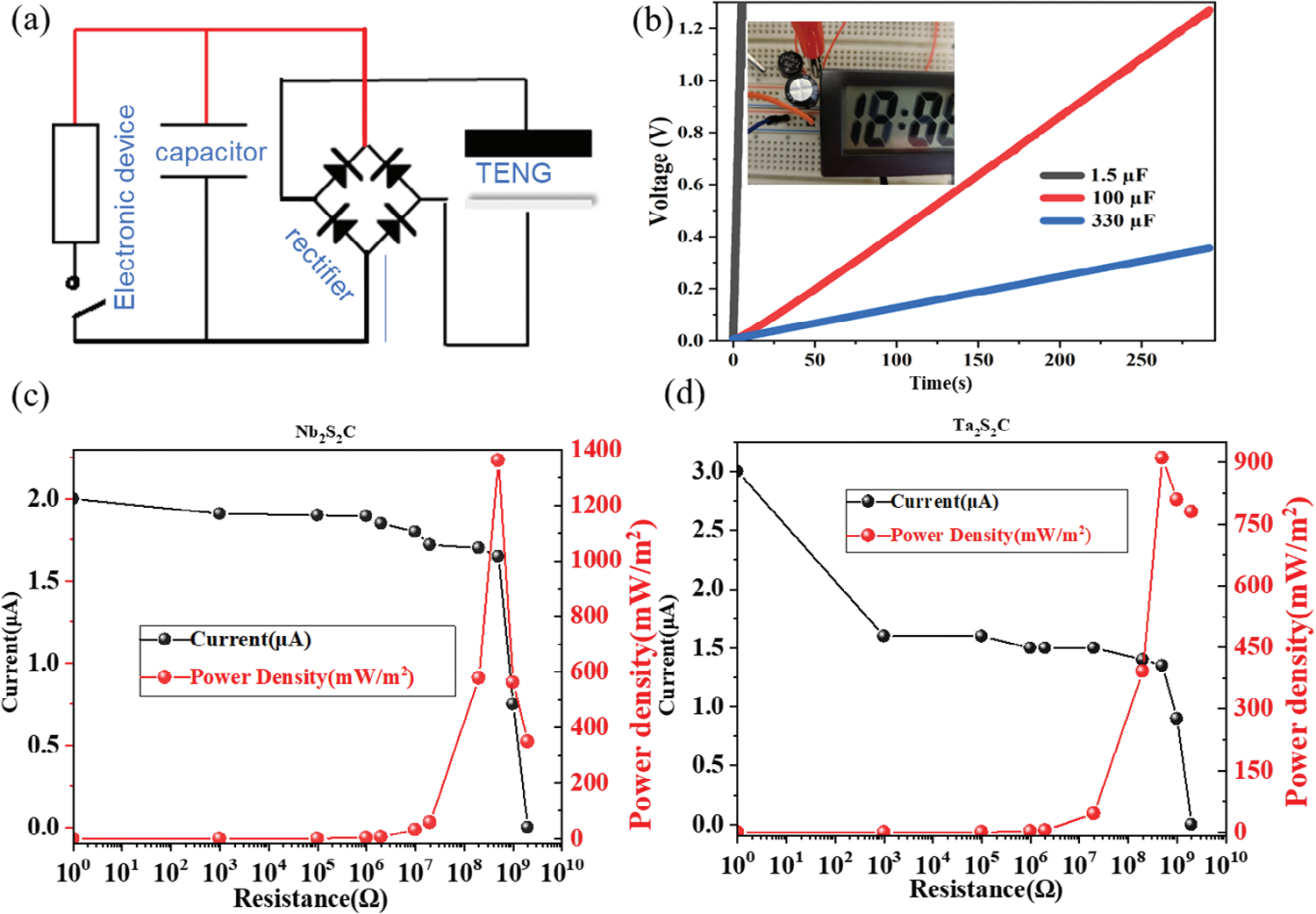
a) Electrical circuit of TENG charging capacitor and powering electronic device. b) The voltage curve of charging capacitors of 1.5, 100, and 330 µF and the inset of (b) is the image of the powering clock by the charged capacitor. c) Current and output power density curve of the Nb_2_S_2_C‐based TENG at different resistances from 1 Ω to 2 GΩ under 20 N and 2 Hz impact. d) Current and output power density curve of the Ta_2_S_2_C‐based TENG at different resistances from 1 Ω to 2 GΩ under 20 N and 2 Hz impact.

With rectifier and capacitor circuit integration (**Figure** **6**a), a capacitor of 100 µF was charged by TENG, and then a calculator or watch could be powered up consistently and stably,^[^ ^60^ ^]^ as shown in Video S2 (Supporting Information), The charging capacity was further evaluated by charging different values of capacitors including 1.5, 100, and 330 µF with charging rate as 220, 4, and 1.4 mV s^−1^, respectively, as shown in Figure 6b. It is worth noting that the triboelectric generator has a high internal impedance output characteristic, making it hard to directly charge batteries.^[^ ^19^, ^20^ ^]^

Under 20 N force and 2 Hz frequency impact with a size of 10 cm^2^, different resistors ranging from 1 kΩ to 2 GΩ were adopted to evaluate the output voltage and current, and then the power density output was calculated accordingly.^[^ ^24^ ^]^ The voltage rises as resistances increase, and by contrast, the current decreases gradually with the increase of resistances (Figure 6c,d). The instantaneous peak power density of the TMCC‐TENG could be calculated by P = I^2^R/S, where P, I, R, and S denote power density, output current, external resistance, and effective size of the TMCC‐TENG, respectively.^[^ ^61^ ^]^ The power density of Nb_2_S_2_C‐based TENG increased with the resistances changed from 1 kΩ to 500 MΩ and reached the maximum of 1360 mW m^−2^ at 500 MΩ load resistor (Figure 6c). Under the same circumstance, the power density of Ta_2_S_2_C‐based TENG also reached a top value of 911 mW m^−2^ with a resistor of 500 MΩ load (Figure 6d). Table S1 (Supporting Information) provides a performance comparison of our as‐made TENGs with other 2D material‐based TENGs recently reported such as graphene, graphene oxide, boron nitride, MXene, g‐C_3_N_4,_ MoS_2_, and it is noteworthy that the power density of TMCC‐TENGs is among the highest level of most reported 2D TENGs.

**Figure 7:**
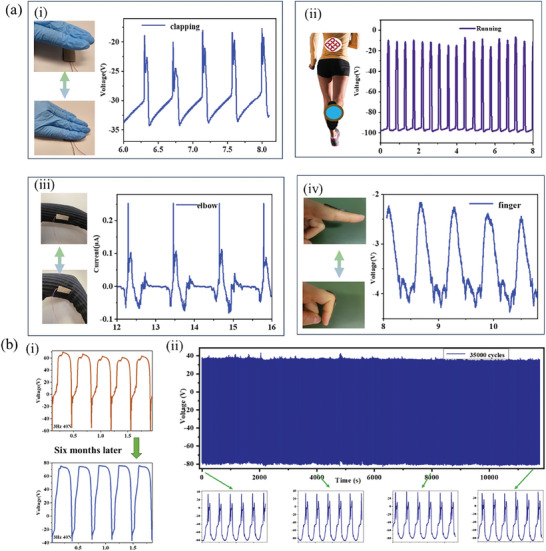
a) Applications of TMCC‐TENG. The output response of wearable harvester and sensor under mechanical movements i) clapping and ii) running. The self‐powered sensing at different positions of the human body iii) elbow and iv) finger bending. b) Durability experiments for TENG i) before and after six months and ii) operated for 35 000 cycles at a frequency of 3 Hz.

**Figure** **7**a shows that TMCC‐TENG can be attached to several parts of the human body for energy harvesting and act as self‐powered human gesture sensors. Mechanical energy could be harvested when one single TMCC‐TENG was attached under the insole while running, generating Voc up to ≈90 V (Figure 7a‐ii), as well as clapping with Voc at 15 V (Figure 7a‐i). In order to study the electric performance of human gesture sensors, TMCC‐TENG were deformed into various shapes and fixed at different positions of the human body to investigate the electrical signal generated while repeated bending, where TMCC‐TENG adhered to elbow exhibited Isc of 0.33 µA (Figure 7a‐iii) and finger exhibited Voc of 2 V, respectively (Figure 7a‐iv).

It is also vital to assess TENG's physical reliability during long‐term operation in actual application scenarios. It is noted that TMCC‐TENG's voltage output remained stable after being exposed to air for up to 6 months (Figure 7b‐i) owing to PDMS's stale physical and chemical nature. Moreover, after 35 000 cycles of continuous contact separation strikes, the amplitude of the triboelectric output signal remained almost consistent (Figure 7b‐ii), demonstrating the excellent durability of TMCC‐TENGs. We have also evaluated the thermal stability and chemical stability for TMCC/PDMS composite, as shown in Figure S6 (Supporting Information), as well as hydrophobicity shown in Figure S7 (Supporting Information).

### 2.5 Tribology Properties

**Figure 8:**
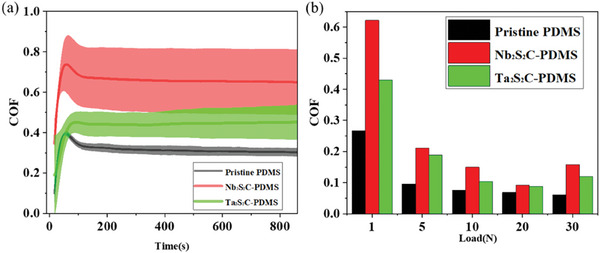
Tribology Test for friction performance of pristine PDMS, Nb_2_S_2_C/PDMS composite, and Ta_2_S_2_C/PDMS composite substrate in ball‐on‐disk experiments a) Derivatives and average COF of 3 times experiments from time 0 s to 850 s at the load of 1 N. b) average COF after data stabilization (150s after the start of the experiment approximately) at the load of 1, 5,10, 20, 30 N, respectively.

To evaluate the friction and wear performance of the fabricated TMCC/PDMS composites as compared to the pristine PDMS samples, we performed reciprocating ball‐on disk experiments^[^ ^62^ ^]^ using a 100Cr6 6 mm diameter steel ball as counter body, as shown in Video S3 (Supporting Information). At a load of 1 N, the average coefficient of friction (COF) quickly increased within the first 100 s of running‐in to a maximum value of 0.71, 0.43, and 0.395 for Nb_2_S_2_C/PDMS composite, Ta_2_S_2_C/PDMS composite, and pristine PDMS, respectively. Subsequently, the COF slightly decreased until it stabilized at a steady‐state value of 0.62, 0.43, 0.28 for Nb_2_S_2_C/PDMS composite, Ta_2_S_2_C/PDMS composite, and pristine PDMS, respectively. The tribological experiments were repeated three times^[^ ^63^ ^]^ at the load of 1 N, and then the corresponding standard deviations (shade area) and mean values were calculated, as shown in **Figure** **8**a. Besides, experiments under other loads including 5, 10, 20, and 30 N were also investigated with the average values shown in the bar graph Figure 8b. Generally, the COF of both TMCC/PDMS composites was higher than that of the pristine PDMS samples, while the COF of the Nb_2_S_2_C/PDMS composite was found to be consistently higher than that of the Ta_2_S_2_C/PDMS composite under different loads. This can be attributed to the evolution of the real contact area, leading to higher adhesion between the friction partners. With an increase in load from 1 to 5 N, the COF of all tested samples drastically drops by more than 50%. The lowest COF for the pristine samples was observed at the maximum load of 30 N, whereas both composites demonstrated the lowest COFs at 20 N from where the COF again increases at 30 N. We anticipated that this is related to the activation of the beneficial tribological properties of the TMCCs with higher loads resulted from their layered structure facilitating easy shear. At the highest load of 30 N, the TMCC might start to degrade during sliding and, therefore lose their beneficial tribological effect.

For the reciprocating ball‐on‐disk system in the tribological experiment of COF, we used 6 mm diameter stainless steel balls (100Cr6) as a counter body with PDMS plates of around 2 mm thickness underneath. Figure S3 (Supporting Information) shows the scratches on the steel ball, pristine PDMS, and micrographs of 10 wt.‰ Nb_2_S_2_C/PDMS composite and 10 wt.‰ Ta_2_S_2_C/PDMS composite after COF experiment under the same conditions. After 1 h of friction under 1 N with the counter body of 100Cr6 steel ball and PDMS substrate, the average depth difference values of ten times of pristine PDMS, Nb_2_S_2_C/PDMS composite, Ta_2_S_2_C/PDMS composite were 9.244, 6.096, 4.708 µm, respectively. In these examinations imitating sliding mode TENG, significant wear tracks were not detected for both TMCC/PDMS composites as shown in Figure S3 (Supporting Information), and nanoindentation or nano scratch experiment can be further experimented with to recognize wear or scratch. Overall, Ta_2_S_2_C/PDMS composite exhibited smaller COF, reduced wear, and softer texture as compared to Nb_2_S_2_C/PDMS composite, while maintaining excellent electrical performance with slightly larger Voc, Isc, and Qsc. The triboelectric performance of TMCC/PDMS was higher than pristine PDMS. The COF of TMCC/PDMS is higher than that of pure PDMS, which means it generates higher friction in sliding situations and might prevent contact‐separation TENG from sliding sideways.

## 3 Conclusion

In summary, two species (Nb_2_S_2_C and Ta_2_S_2_C) of a new family of 2D materials, Transition Metal Carbo‐Chalcogenides (TMCCs), were first employed to develop new TENGs with doping with PDMS. The optimum concentration ratio of 3 wt. ‰ was identified to achieve the best electric performance. The maximum power density of 1 360 and 911 mW m^−2^ could be reached for Nb_2_S_2_C and Ta_2_S_2_C‐based TENG respectively. The influence of impact frequency and force were also studied. Moreover, the tribology evaluation revealed that the Ta_2_S_2_C/PDMS composite presented a lower coefficient of friction (COF) than Nb_2_S_2_C/PDMS composite. The as‐made TENGs could perform as a sustainable power source for charging capacitors and driving small electronics, as well as perform as self‐power sensors for the detection of human movements. In the future, the TMCC‐TENG may play a useful role in human–machine interaction and large‐scale energy harvesting for sustainable and renewable energy applications.

## 4 Experimental Section

### Materials

PDMS (SYLGARD 184 Silicone Elastomer kit) was purchased from Dow Corning Co, Ltd. Copper (Cu) wire and Aluminum foil were bought from DongguaYishengxing Copper and Aluminum Materials Co, Ltd, China. Cu/Ni conductive fabric (CNF) was purchased from 3M Corp. TMCCs (Nb_2_S_2_C and Ta_2_S_2_C powder) were synthesized from the laboratory of Michael Naguib. All reagents were used as received without further purification.

### Characterization and Measurement

Field Emission Scanning Electron Microscope (SEM, Tescan MIRA) was used to characterize the morphology and Energy Disperse Spectroscopy (EDS) data was acquired simultaneously. X‐ray diffraction (XRD) pattern with scanning from 5 to 70° (2θ) was recorded on an X‐ray diffractometer (Rigaku SmartLab) to identify the crystalline phase. XPS spectra were investigated on an ESCALAB210 spectrometer. Fourier transforms infrared (FTIR) absorption spectra were recorded on Spectrum 100, Perkin Elmer. The dielectric constant was measured using a precision LCR meter (Keysight E4980A). The triboelectric performance measurements under cyclic contact‐separation motion with adjustable frequencies were realized by a life test machine (ZX‐A03, Zhongxingda, Shenzhen) with impact force signal monitored simultaneously by the DAQ (Dewetron, Dewe‐2600 DAQ system). An oscilloscope (Keysight Infiniivision DSOX3024T) was adopted to measure the voltage, while an electrometer (Keithley 6514, Tektronix) was operated to record short‐circuit current and short‐circuit charge transfer. The coefficient of Friction was investigated by the Rtec Tribometer Instrument. KEYENCE 3D Laser Scanning Microscope was employed to measure wear track, scratch, and depth and produce 3D microscopic images. The simulation was conducted with COMSOL Multiphysics software. The kelvin probe force microscopy (KPFM) images were obtained with an atomic force microscope (MFP‐3D Infinity, OXFORD instruments) by using a Pt‐coated tip.

### Fabrication

The TMCC‐TENGs were prepared by the blade coating method, as illustrated in Figure 1a,b. TMCC was added at a certain weight ratio into PDMS and ultrasonicated for more than 12 h for thorough distribution and dispersion, subsequently the curing agent with 1/9 the weight of PDMS liquid was added into the mixture and stirred for 20 min, then blade coating the liquid to film with a thickness of ≈2mm, finally put in an oven at 80° for 12 h until completely cured. PDMS solution was cured by heat and the TMCC particles thereby were mixed and dispersed uniformly and fixed in the cured PDMS matrix. To quantify the triboelectric performance, TMCC‐TENGs were assembled as follows: the TMCC/PDMS composite was adhered to Cu/Ni conductive fabric (CNF) to form the negative part, and the positive part was only CNF acting as both triboelectric material and electrode or Nylon as tribopositive material and adhered to CNF as an electrode. The CNF electrodes were connected through copper wire and then to the electrometer to measure and collect signal of open‐circuit voltage (Voc) and short‐circuit current (Isc) as well as short‐circuit charge transfer (Qsc).

### Tribology Evaluation

A ball‐on‐disk tribometer in linear reciprocating sliding (Rtec Instruments) was used to test the frictional properties of the composites using a stroke length of 1 mm, a linear sliding velocity of 1 mm s^−1^, an acceleration of 0.1 s (1cm s^−2^), and normal loads of 1, 5, 10, 20, and 30 N. The load of 1 N corresponds to a Hertzian contact pressure of around 0.5 MPa. Stainless steel balls (100Cr6) with a diameter of 6 mm were used as counter bodies against the pristine PDMS plate and the composite plates. The tribological experiments were repeated three times for statistical representation, and the curves were used to calculate the corresponding mean values and standard deviations.

### Finite Element Simulation Through COMSOL

The Electrostatics Interface module of COMSOL was used to model the operation of TENG in contact‐separation mode. The model was made with the same thickness (1 mm) and width (40 mm) as the experimental samples. The contact‐separation behaviors were realized by the parametric sweep investigation with a separation distance of 10 mm. The simulations of the potential distribution correspond at various separation distances. The simulated open‐circuit voltage was around 170 V while the measured values were around 120 V. The simulation results confirm the TMCC/PDMS TENG's operational concept, as shown in Figure S5 and Video S4 (Supporting Information).

## Conflict of Interest

The authors declare no conflict of interest.

## Supporting information



Supporting Information

Supplemental Video 1

Supplemental Video 2

Supplemental Video 3

Supplemental Video 4

## Data Availability

The data that support the findings of this study are available from the corresponding author upon reasonable request.
