# Supplementary material for: Triboelectric Nanogenerators Based on Transition Metal Carbo‐Chalcogenide (Nb2S2C and Ta2S2C) for Energy Harvesting and Self‐Powered Sensing
Source: Adv Sci (Weinh). 2024 Sep 25;11(43):2409619. doi: 10.1002/advs.202409619 (PMC11578342; doi:10.1002/advs.202409619)
Supplement: Supplementary file 1 — Supporting Information [file ADVS-11-2409619-s004.docx]

**Supporting Information**


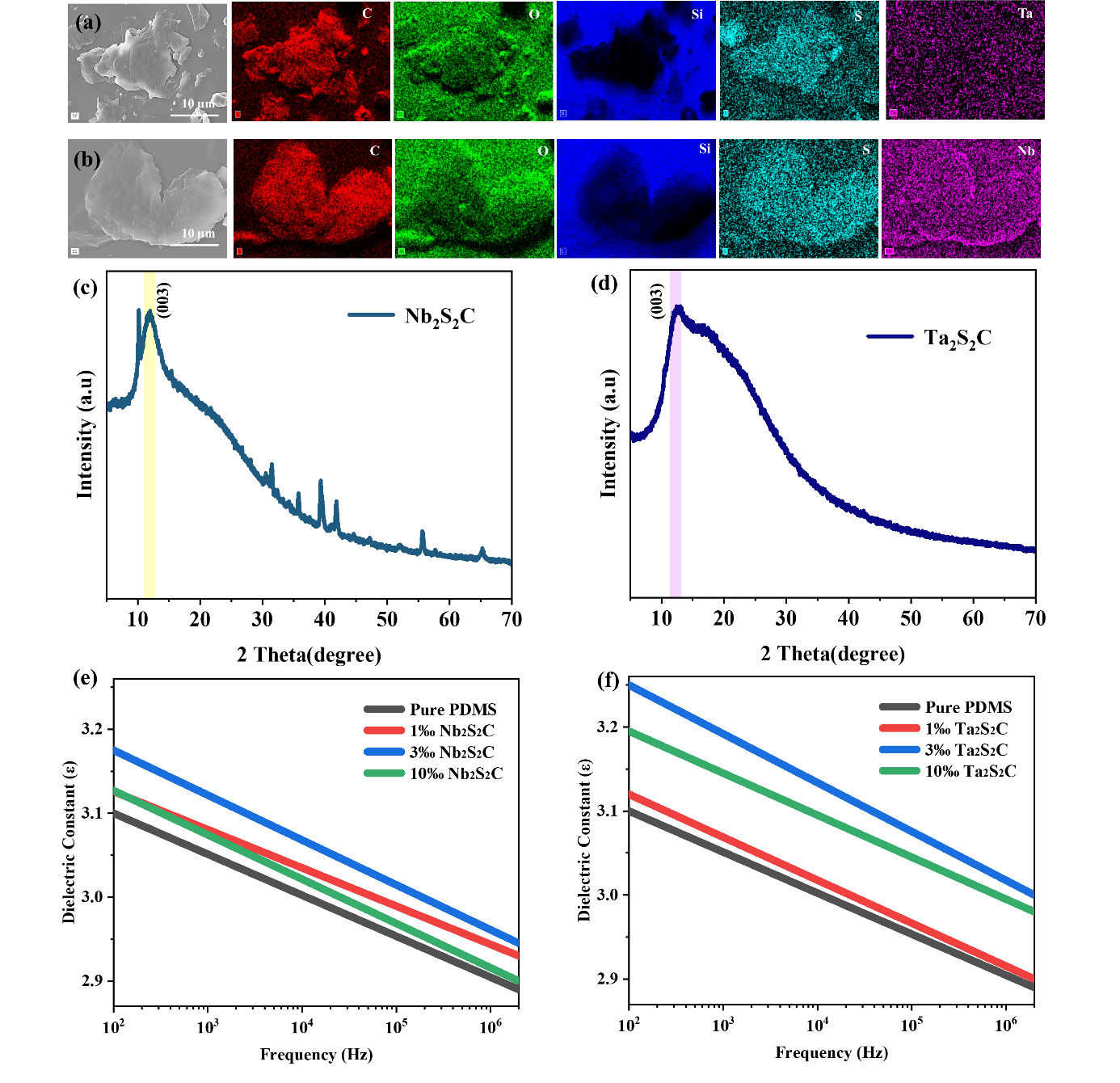


Figure S1. Morphology and elemental analysis (a) EDS-elemental mapping images of the Nb_2_S_2_C/PDMS composite. (b) EDS-elemental mapping images of the Ta_2_S_2_C/PDMS composite. (c) XRD Pattern of Nb_2_S_2_C/PDMS composite. (d) XRD Pattern of Ta_2_S_2_C/PDMS composite. (e) Frequency dependence of dielectric constant of Nb_2_S_2_C/PDMS composite with different concentrations of 0, 1 wt. ‰, 3 wt. ‰, and 10 wt. ‰. (f) Frequency dependence of dielectric constant of Ta_2_S_2_C/PDMS composite with different concentrations of 0, 1 wt. ‰, 3 wt. ‰, and 10 wt. ‰.


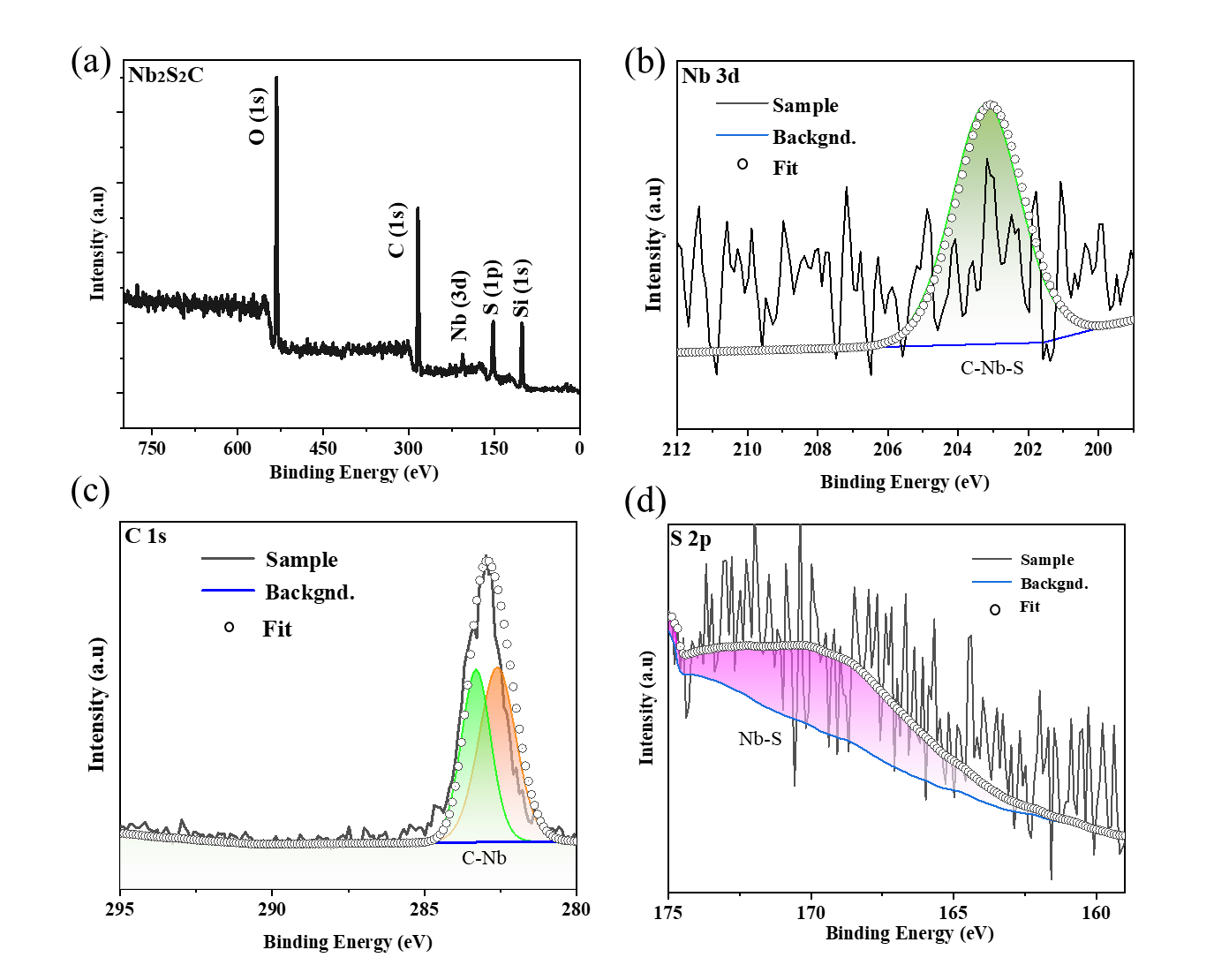

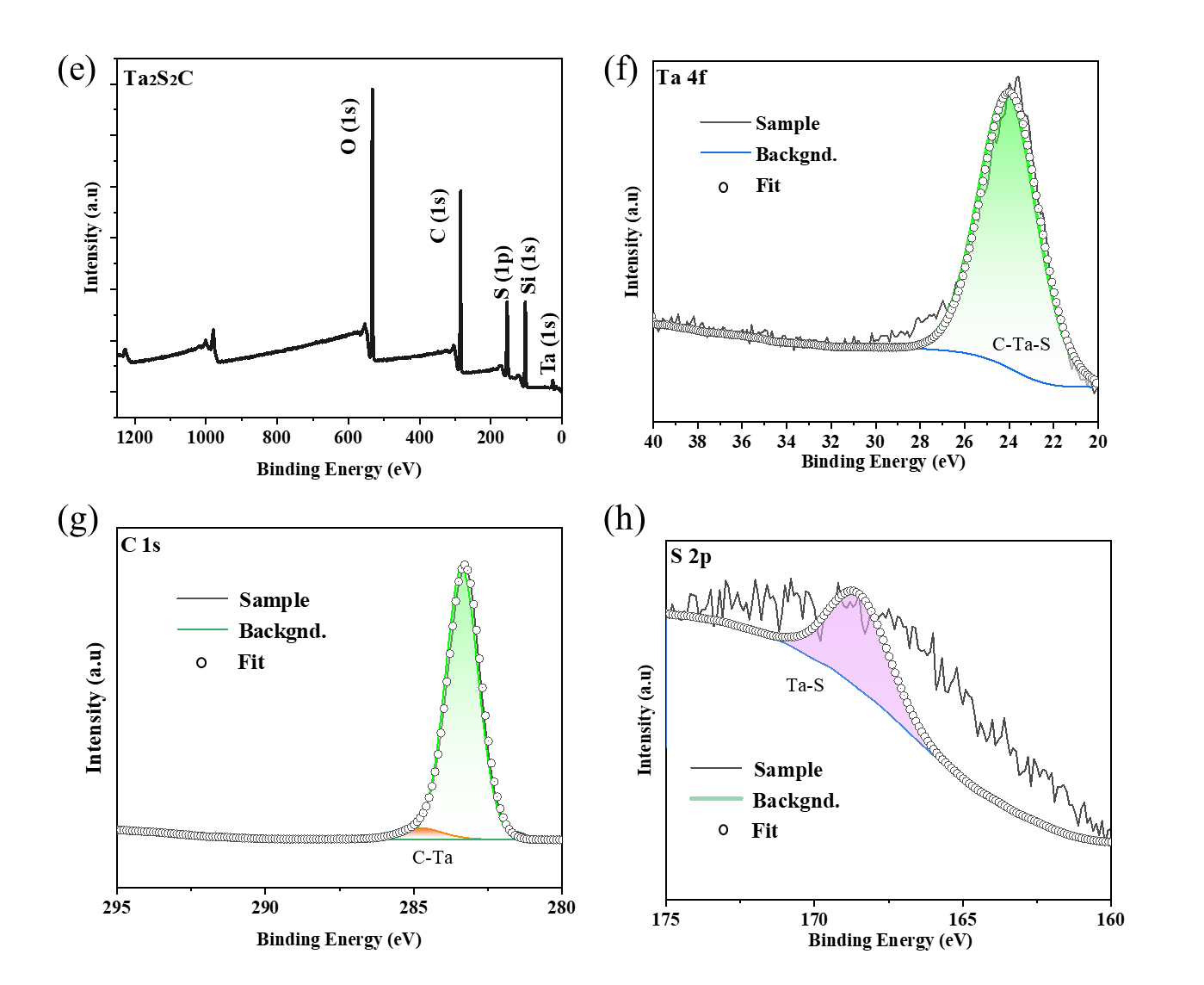


Figure S2. XPS spectra of TMCC/PDMS composites. (a) Nb_2_S_2_C, (b) Nb 3d, (c) C 1s, (d) S 2p. (e)Ta_2_S_2_C. (f) Ta 4f, (g) C 1s, (h) S 2p.


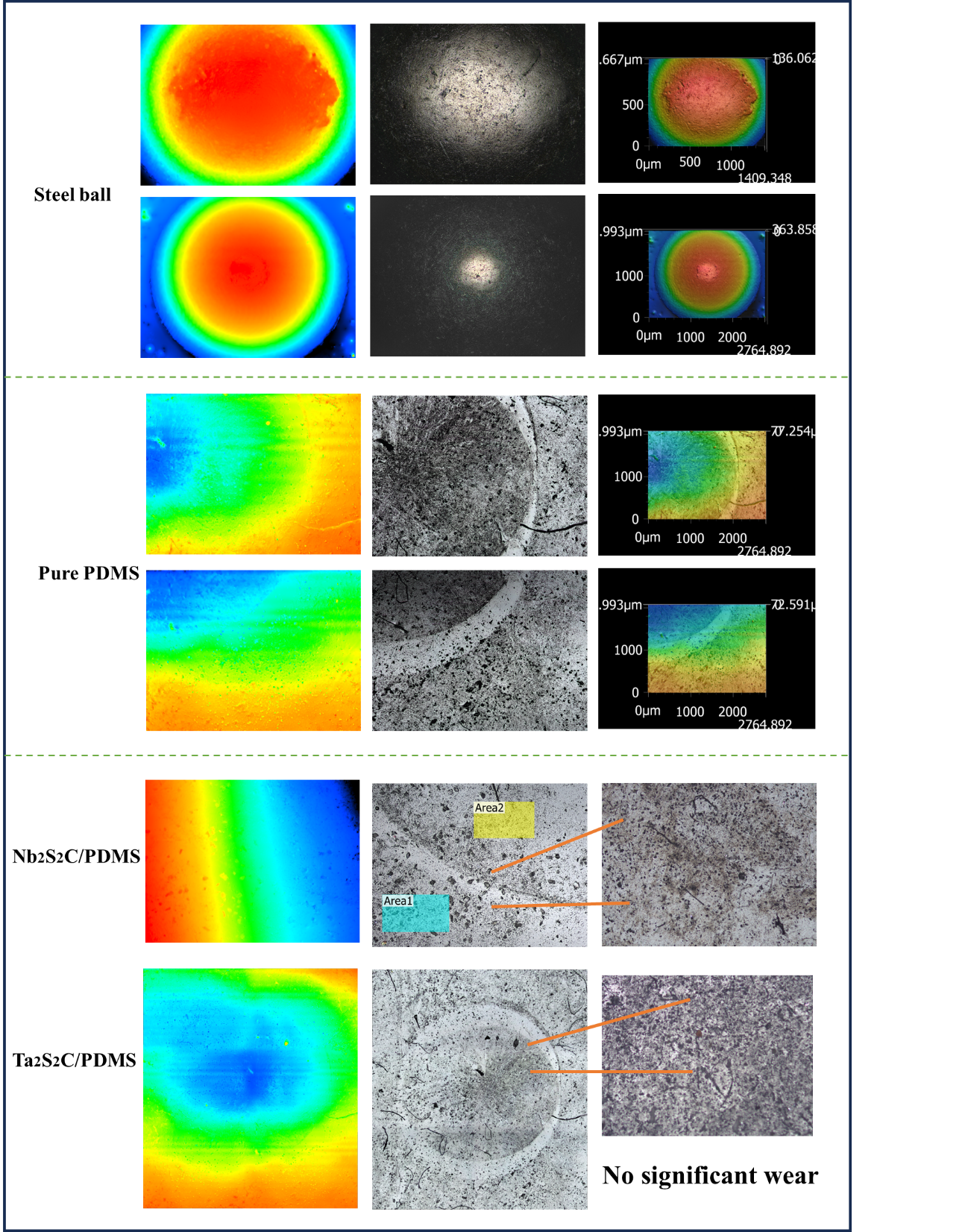


Figure S3. Depth difference and SEM Micrograph of the steel ball and pristine PDMS, Nb_2_S_2_C/PDMS composite, and Ta_2_S_2_C/PDMS composite after consistent ball-on-disk experiment for one hour under 1N.

Table S1. Performance comparison of our device with other two-dimensional materials based TENGs recently reported

| **Negative Layer (Dopants@)** | **Positive Layer** | **V**  **(V)** | **I**  **(μA)** | **Power density**  **(**mW/m^2^) | **Ref.** |
| --- | --- | --- | --- | --- | --- |
| PDMS | AgNPs/MXene ink fibre | 7.7 | 7 | / | 1 |
| Graphene | PET | 5 | 0.5 | / | 2 |
| Graphene Nanosheets/PTFE | Al | 96 | 3.66 | 390 | 3 |
| Silicone | Crumpled Graphene | 9.3 | / | 15 | 4 |
| PTFE | g-C_3_N_4_@PA_66_ | 80 | 6 | 45 | 5 |
| Al_2_O_3_/Hexagonal Boron Nitride | Graphene | 1.2 | / | / | 6 |
| Polyimide/Boron Nitride Nanosheet | Al | 65.9 | 4.5 | 214 | 7 |
| PDMS | MoS_2_/SiO_2_ | 25 | 1.2 | 22.5 | 8 |
| LC-WS_2_ | PET | 12.2 | / | 138 | 9 |
| Crumpled Mxene | Skin | 16.4 | 2.67 | 28.9 | 10 |
| Carbonized MXene/ZiF-67 | hand | 35 | 12.5 | 55 | 11 |
| Mg-Al Layered Double Hydroxides | water droplet | 13 | 1.6 | / | 12 |
| Kapton/PI | rGO | 90 | 6.3 | / | 13 |
| Nb_2_S_2_C@PDMS | Nylon | 112 | 8.6 | 1,360 | this work |
| Ta_2_S_2_C@PDMS | Nylon | 130 | 9.2 | 911 | this work |


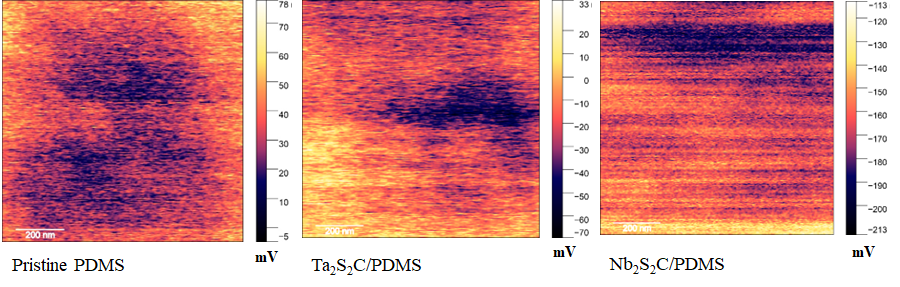


Figure S4. Kelvin probe force microscopy (KPFM) images of the pristine PDMS, Ta_2_S_2_C/PDMS and Nb_2_S_2_C/PDMS, composite films, respectively.


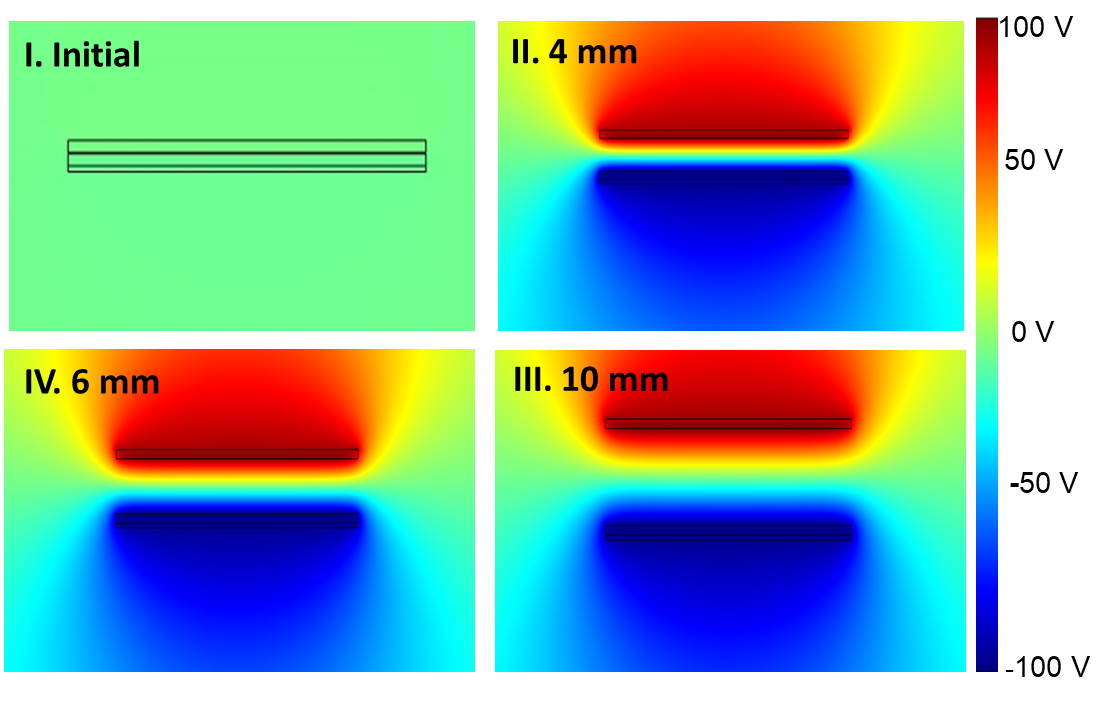


Figure S5. The COMSOL simulation results of the potential distributions for the TMCC/PDMS TENG under vertical contact-separation mode (Unit: Voltage).


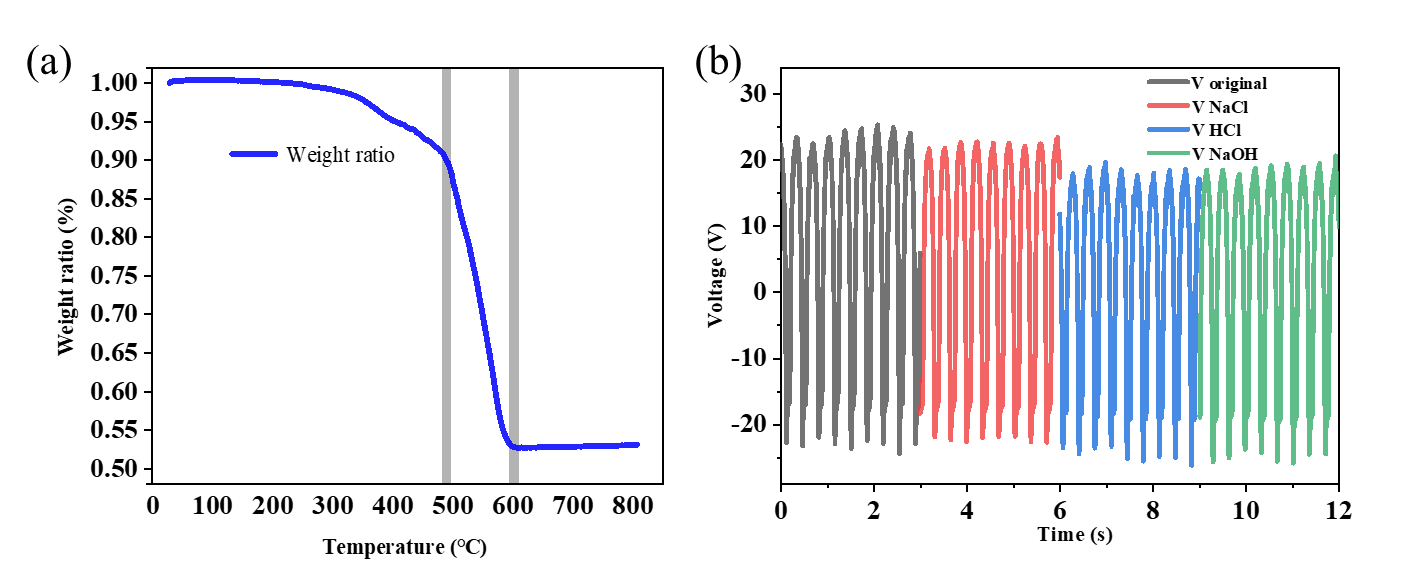


Figure S6. (a) TGA plots of TMCC/PDMS. (b) Open-circuit voltage of 4 equal pieces of TMCC-TENG, with the TMCC/PDMS composite immersed after 24 h in normal air, concentrated NaCl, HCl, NaOH solutions, respectively.


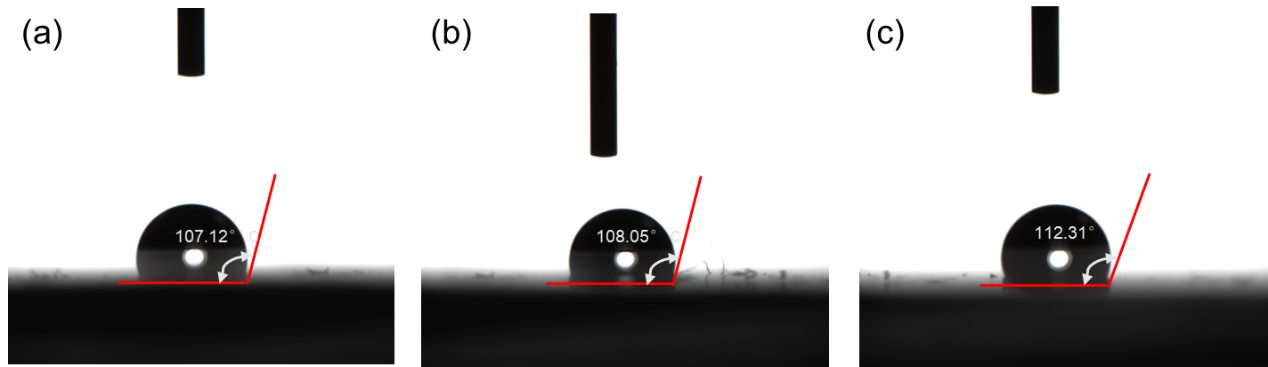


Figure S7. Contact angle (a) pristine PDMS 107.12° (b) Nb_2_S_2_C/PDMS composite 108.05°, and (c) Ta_2_S_2_C/PDMS composite 112.31°.

Reference:

1. Jiang, C. M.; Li, X. J.; Ying, Y. B.; Ping, J. F., A multifunctional TENG yarn integrated into agrotextile for building intelligent agriculture. *Nano Energy* **2020,** *74,*104863.

2. Kim, S.; Gupta, M. K.; Lee, K. Y.; Sohn, A.; Kim, T. Y.; Shin, K. S.; Kim, D.; Kim, S. K.; Lee, K. H.; Shin, H. J.; Kim, D. W.; Kim, S. W., Transparent flexible graphene triboelectric nanogenerators. *Adv. Mater.* **2014,** *26* (23), 3918-3925.

3. Yang, P.; Wang, P.; Diao, D., Graphene Nanosheets Enhanced Triboelectric Output Performances of PTFE Films. *ACS Appl. Electron. Mater.* **2022,** *4* (6), 2839-2850.

4. Chen, H.; Xu, Y.; Bai, L.; Jiang, Y.; Zhang, J.; Zhao, C.; Li, T.; Yu, H.; Song, G.; Zhang, N.; Gan, Q., Crumpled Graphene Triboelectric Nanogenerators: Smaller Devices with Higher Output Performance. *Adv. Mater. Technol.* **2017,** *2* (6), 1700044.

5. Xiao, Y.; Xu, B.; Bao, Q.; Lam, Y., Wearable Triboelectric Nanogenerators Based on Polyamide Composites Doped with 2D Graphitic Carbon Nitride. *Polymers* **2022,** *14* (15), 3029.

6. Han, S. A.; Lee, K. H.; Kim, T.-H.; Seung, W.; Lee, S. K.; Choi, S.; Kumar, B.; Bhatia, R.; Shin, H.-J.; Lee, W.-J.; Kim, S.; Kim, H. S.; Choi, J.-Y.; Kim, S.-W., Hexagonal boron nitride assisted growth of stoichiometric Al2O3 dielectric on graphene for triboelectric nanogenerators. *Nano Energy* **2015,** *12*, 556-566.

7. Pang, L.; Li, Z.; Zhao, Y.; Zhang, X.; Du, W.; Chen, L.; Yu, A.; Zhai, J., Triboelectric Nanogenerator Based on Polyimide/Boron Nitride Nanosheets/Polyimide Nanocomposite Film with Enhanced Electrical Performance. *ACS Appl. Electron. Mater.* **2022,** *4* (6), 3027-3035.

8. Park, S.; Park, J.; Kim, Y.-g.; Bae, S.; Kim, T.-W.; Park, K.-I.; Hong, B. H.; Jeong, C. K.; Lee, S.-K., Laser-directed synthesis of strain-induced crumpled MoS_2_ structure for enhanced triboelectrification toward haptic sensors. *Nano Energy* **2020,** *78,* 105266.

9. Kim, T. I.; Park, I. J.; Kang, S.; Kim, T. S.; Choi, S. Y., Enhanced Triboelectric Nanogenerator Based on Tungsten Disulfide via Thiolated Ligand Conjugation. *ACS Appl. Mater. Interfaces* **2021,** *13* (18), 21299-21309.

10. Cao, Y.; Guo, Y.; Chen, Z.; Yang, W.; Li, K.; He, X.; Li, J., Highly sensitive self-powered pressure and strain sensor based on crumpled MXene film for wireless human motion detection. *Nano Energy* **2022,** *92,* 106689.

11. Salauddin, M.; Rana, S. M. S.; Sharifuzzaman, M.; Lee, S. H.; Zahed, M. A.; Do Shin, Y.; Seonu, S.; Song, H. S.; Bhatta, T.; Park, J. Y., Laser-carbonized MXene/ZiF-67 nanocomposite as an intermediate layer for boosting the output performance of fabric-based triboelectric nanogenerator. *Nano Energy* **2022,** *100,* 107462 .

12. Cui, P.; Wang, J.; Xiong, J.; Li, S.; Zhang, W.; Liu, X.; Gu, G.; Guo, J.; Zhang, B.; Cheng, G.; Du, Z., Meter-scale fabrication of water-driven triboelectric nanogenerator based on in-situ grown layered double hydroxides through a bottom-up approach. *Nano Energy* **2020,** *71,* 104646.

13. Wu, C.; Kim, T. W.; Choi, H. Y., Reduced graphene-oxide acting as electron-trapping sites in the friction layer for giant triboelectric enhancement. *Nano Energy* **2017,** *32*, 542-550.
