# Supplementary figures and images for: Triboelectric Nanogenerators Based on Transition Metal Carbo‐Chalcogenide (Nb2S2C and Ta2S2C) for Energy Harvesting and Self‐Powered Sensing
Source: Adv Sci (Weinh). 2024 Sep 25;11(43):2409619. doi: 10.1002/advs.202409619 (PMC11578342; doi:10.1002/advs.202409619)

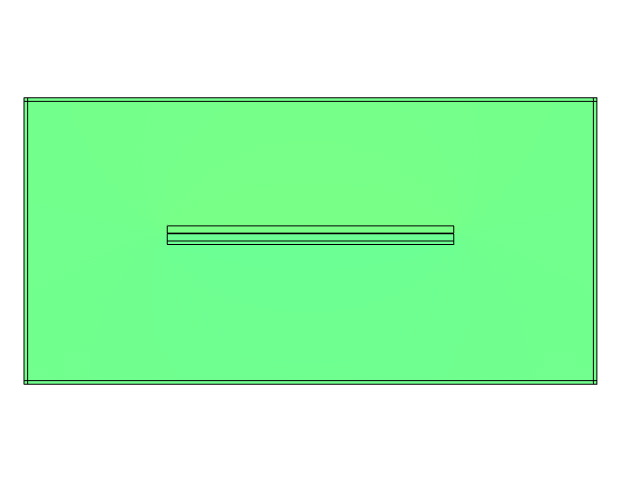

Supplement: Supplementary file 5 — Supplemental Video 4 [file ADVS-11-2409619-s001.gif]
